# An analysis of the distribution and spectrum of alpha thalassemia mutations in Rasht City, North of Iran

**DOI:** 10.3389/fped.2023.1039148

**Published:** 2023-03-22

**Authors:** Mona Asghari Ahmadabad, Noushin Pourreza, Setareh Ramezanpour, Adel Baghersalimi, Mersedeh Enshaei, Marjan Askari, Amirhossein Alizadeh, Elahe Izadi, Bahram Darbandi

**Affiliations:** ^1^ Pediatric Department, Pediatric Diseases Research Center, 17 Shahrivar Children's Hospital, School of Medicine, Guilan University of Medical Sciences, Rasht, Iran; ^2^ Department of Genetic Disorders,Razi Pathobiology and Genetics Laboratory, Rasht, Iran

**Keywords:** alpha thalassemia, genetic mutation, genetic diagnosis, molecular spectrum, Rasht, Guilan province, Iran

## Abstract

**Background:**

Alpha thalassemia is one of the most common hereditary hemoglobin disorders worldwide, particularly in the Middle East, including Iran. Therefore, determining the spectrum and distribution of alpha thalassemia mutation is a fundamental component of preventive approaches and management strategies.

**Methods:**

The present study reviews the genetic testing and blood laboratory results of 455 candidates eligible for marriage who were suspected of being thalassemia carriers and on whom genetic testing was performed from 21 March 2013 to 31 December 2020 in Rasht City.

**Results:**

A total of 114 (25.05%) alpha thalassemia cases were identified. Fifteen different alpha mutations were found. The most common mutation among the study population was −α^3.7^ deletion in 55 patients (48.24%), followed by Hb Constant Spring (C.S) in 21 patients (18.42%) and poly A2 in 16 (14.03%). Also, most of the patients were silent carriers. The deletion type of mutation was much more common than non-deletion mutations.

**Conclusion:**

Our study reveals genetic heterogeneity and alpha thalassemia diversity among the Rasht City population. We expect that these findings will help guide premarital screening and genetic counseling, prenatal diagnosis of thalassemia, preventive strategy development, as well as a compilation of the alpha thalassemia catalog in Guilan province.

## Introduction

Hemoglobin disorders are a health issue in countries with high birth rates worldwide (1). Alpha thalassemia is an inherited, autosomal recessive disorder in which alpha-globin gene expression is suppressed or reduced and is characterized by microcytic hypochromic anemia. It is one of the most common monogenic gene disorders (2). The most affected individuals present variable degrees of anemia, reduced mean corpuscular hemoglobin (MCH) and mean corpuscular volume (MCV), as well as an average to a slightly decreased level of hemoglobin A2 (HbA2) (3).

The HBA1 and HBA2 genomes are located on chromosome 16 (16p13.3) and encoded functional alpha genes (α_2_α_1_/α_2_α_1_) in the human diploid genome, which are responsible for the production of alpha-globin chains (4, 5). According to previous studies, gene deletion accounted for more than 95% of alpha thalassemia cases and was followed by point mutations (6). The absence of both genes on a chromosome is denoted as α^0^ alleles, while a partial deletion of α_1_ and/or α_2_ is denoted as α^+^ and leads to decreased α-globin chain synthesis (7). The absence of one gene (−α/αα) causes the silent α-thal carrier, and the absence of two genes (−/αα, −α/−α) causes the α-thal trait that results in mild hypochromic microcytic anemia. Also, three-gene deletion (−/−α) generates Hemoglobin H disease (Hb H), which is associated with moderate to severe anemia. Thus, Hb Barts hydrops fetalis (ϒ₄) is produced by deleting all four genes and causes a fatal situation (8). The −α^3.7^ mutation is the most prevalent (43.84%), followed by the α^IVS−1/(−5NT)^ with a prevalence rate of 4.91%. The less-frequent mutations are Hb ICARIA and α codon16 (9).

The Iranian National Thalassemia Screening Program has been successful in significantly decreasing thalassemia major infant birth rates during the past two decades. Still, as a part of the “thalassemia belt,” Iran is a country with a very high rate of thalassemia carriers (10). Since Iran has a large population representing multiple ethnic groups, we need to determine the distribution of the α-globin gene mutation across the country. Therefore, this study aims to investigate the spectrum and distribution of alpha thalassemia mutations among candidates eligible for marriage in Rasht city, who were subjected to genetic testing from 2013 to 2020.

## Materials and methods

### Study design and data collection

This retrospective study was conducted at a referral premarital screening health center in Rasht. Based on the latest edition of the thalassemia screening program, all candidates of marriageable age must be referred to the health center for the purpose of obtaining a premarital certificate. Individuals of all age groups who were subjected to genetic testing were enrolled in this study from 21 March 2013 to 31 December 2020. Firstly, we retrieved the medical records of health centers to provide a list of patients on whom genetic testing was performed. Secondly, the genetic testing results were reviewed.

Candidates were selected for this study if they met the following criteria: (1) should be an Iranian citizen and (2) genetic testing results were available. Exclusion criteria included (1) patients who came from outside the province and (2) those with incomplete medical files.

**Figure 1:**
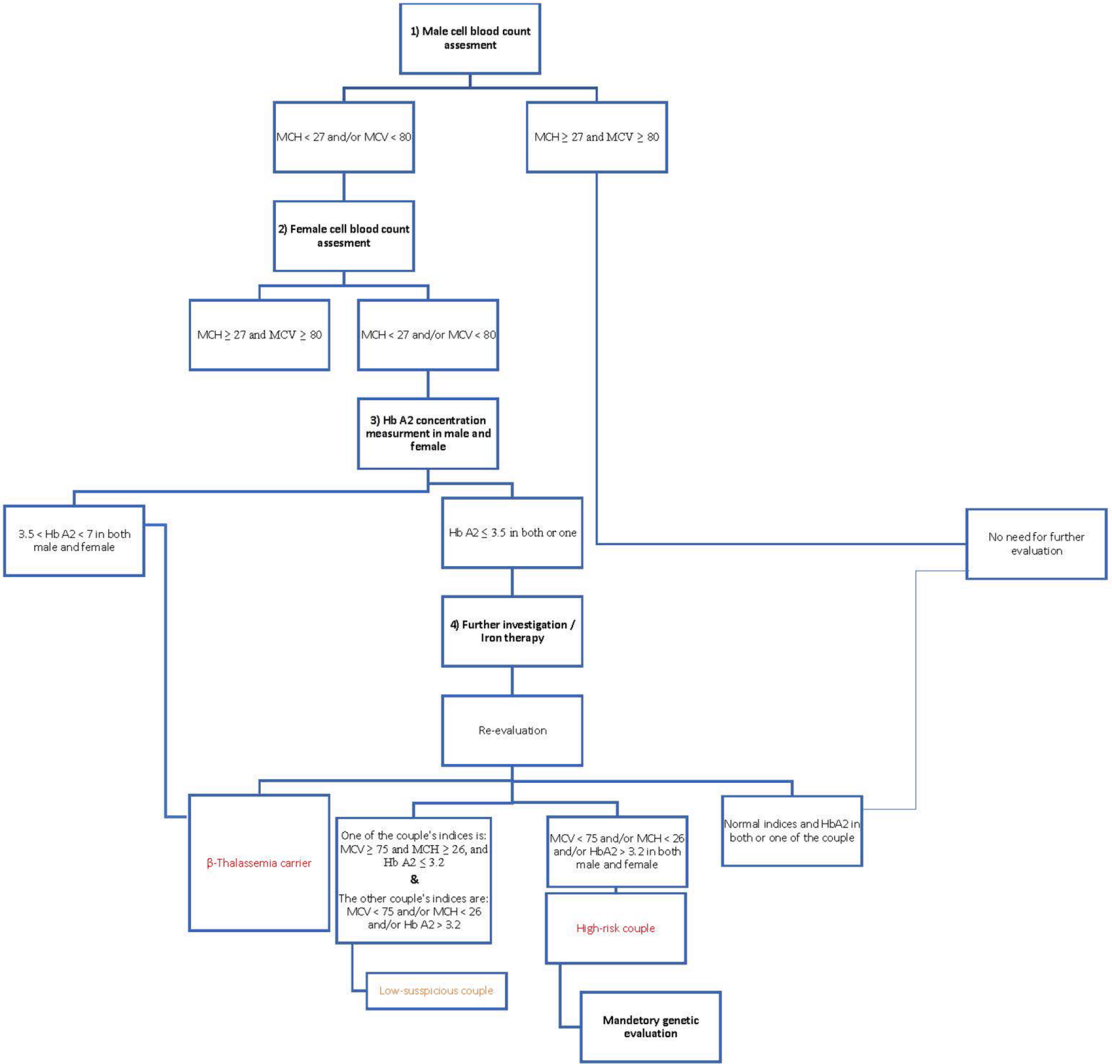
.

According to the Iranian National Thalassemia Screening Program, male blood indices are checked in the first step, and if the person has microcytosis and/or hypochromic anemia (MCV < 80 and/or MCH < 27), female blood indices are also checked. When both have microcytosis and/or hypothermia, HbA2 concentration is measured, and if the level is higher than 3.5%, it becomes a diagnostic criterion for the beta-thalassemia trait. Individuals with HbA2 ≤ 3.5 underwent iron therapy for 1–3 months; then, their blood indices were rechecked; if the indices were not corrected, they were referred for genetic evaluation (Figure 1).

To ascertain the cell blood count, 2 mL of venous blood samples in an ethylene diamine tetraacetic acid (EDTA)-containing tube were obtained from individuals; hematological parameters were evaluated using the KX21N Sysmex device, and high-performance liquid chromatography (HPLC) was carried out to measure the HbA2 level.

A DNA study was performed in the genetic laboratory to detect gene mutation using gap-PCR and Sanger sequencing. Also, Multiplex Ligation-dependent Probe Amplification (MLPA) was performed if necessary [first-step prenatal diagnosis (PND)].

Altogether, 814 couples underwent genetic testing from 21 March 2013 to 21 December 2020; 1,140 patients had met the minor-β thalassemia criteria (MCV < 80 or MCH < 27 and 3.5 < HbA2 < 7), and 439 of them were suspected to be low-risk individuals (26 ≤ MCH < 27 and 75 ≤ MCV < 80 and HbA2 ≤ 3.2).

The medical files of all marriageable candidates referred to Health Center No. 5 in Rasht for the purpose of obtaining marital certificates and performing genetic tests were reviewed to fulfill the inclusion criteria distinctly. Data and information on the type of genetic mutation, sex, MCV, MCH, Hb A2, and Hb were extracted using their case number code in the Health Center’s registry system. In the end, 455 candidates met all inclusion criteria.

### Ethical aspect

This study was first approved by the Pediatrics Research Center of the 17 Shahrivar Hospital of the Guilan University of Medical Sciences. Also, the Ethics Research Committee of the Guilan University of Medical Sciences approved it with the code number IR.GUMS.REC.1396.595. We accessed patients’ medical records in the study without revealing their names and personal information. Also, we reviewed the data retrospectively, so that our study did not impact patient diagnosis or management.

### Statistical analysis

The collected data were statistically analyzed using IBM SPSS Statistics for Windows, version 26.0. Mean, maximum, minimum, and standard deviation were used to describe quantitative variables (Hb and MCV), and frequency and percentage were used to describe qualitative variables (genetic mutations).

## Results

Among the 455 patients who were referred for genetic testing, 114 (25.07%) with alpha thalassemia mutations were discovered, with 64 (56.14%) men and 50 (43.86%) women. As shown in Table 1, 3.7 single-gene deletion was the most prevalent mutation that was identified in 55 patients (48.24%), followed by Hb Constant Spring (C.S) in 21 patients (18.42%), and poly A_2_ in 16 (14.03%).

**Table 1:** Frequency and prevalence of the genotype, phenotype, and mutation type of patients.

Genotype	HGVS nomenclature	Phenotype	Mutation type	Prevalence (%)	Frequency
−α^3.7^/αα	NG_000006.1:g.34164_37967del3804	Silent carrier	Deletion	9.45	43
−α^4.2^/αα	NC_000016.10:g.169818_174075del	Silent carrier	Deletion	1.1	5
− −^MED^/αα	NG_000006.1:g.(23641_23662)_(41183_41203)del	α-Thal trait	Deletion	0.88	4
−(α)^20.5^/αα	NG_000006.1:g.(18148_18200)_(37868_37901)del	α-Thal trait	Deletion	0.44	2
αC.301-24delinsCTCGGCCC/αα	HBA2:c.301-24delinsCTCGGCCC	Silent carrier	Deletion	0.44	2
			**Total**		**56**
α^C.S^ α/αα	HBA2:c.427T > C	Silent carrier	Non-deletion	3.74	17
α^poly AII^ α/αα	HBA2:c.*92A > G	Silent carrier	Non-deletion	3.08	14
α^−5nt^ α/αα	HBA2:c.95 + 2_95 + 6delTGAGG	Silent carrier	Non-deletion	0.88	4
α^Codon 19^ α/αα		Silent carrier	Non-deletion	0.66	3
α HbICaria α/αα	HBA2:c.427T > A	Silent carrier	Non-deletion	0.44	2
α^poly AI^ α/αα	HBA2:c.*93_*94delAA	α-Thal trait	Non-deletion	0.22	1
αCodon 99 Lys > Stop/αα	HBA1:c.298A > T	Silent carrier	Non-deletion	0.22	1
α α^IVSI−4A > G^/αα	HBA1:c.95 + 4A > G	Silent carrier	Non-deletion	0.22	1
α^Codon 22^ α/αα	HBA2:c.69C > T	Silent carrier	Non-deletion	0.22	1
			**Total**		**44**
−α^3.7^/α^poly AII^ α		α-Thal trait	Non-deletion/non-deletion	0.44	2
α^poly AII^ α/α^C.S^ α		α-Thal trait	Non-deletion/non-deletion	0.22	1
α^C.S^ α/α^C.S^ α		α-Thal trait	Non-deletion/non-deletion	0.22	1
−α^3.7^/−α^3.7^		α-Thal trait	Deletion/Deletion	1.76	8
−α^3.7^/α^C.S^ α		α-Thal trait	Deletion/non-deletion	0.44	2
			**Total**		**14**
**Total**				**25.07**	**114**

Fifteen different alpha gene mutations were found. The total mean of Hb was 13.58 ± 1.39 (g/dL), MCV was 76.10 ± 5.05 (fL), MCH was 24.47 ± 2.10 (pg/cell), and Hb A_2_ was 2.72 ± 1.23. Table 2 shows red blood indices and Hb A_2_ variations distinctly for each alpha mutation type.

**Table 2:** Red blood cell indices and Hb A_2_ (mean ± SD) of alpha thalassemia gene mutations.

	*N*	Hb A_2_ (%) (mean ± SD)	MCV (fL) (mean ± SD)	Hb (g/dL)	MCH (pg/cell)
α^0^	93	2.6 ± 0.79	77.34 ± 4.03	13.70 ± 1.40	24.90 ± 1.67
α^+^	21	2.77 ± 1.78	70.62 ± 5.73	13.18 ± 1.29	22.47 ± 2.67

Based on the genetic test results, 93 patients (81.58%) were found to be silent carriers (α^0^), and 21 (18.42%) had the alpha thalassemia trait (α^+^). The most common type of mutation was deletion, detected in 56 patients (49.12%), while non-deletion was traced in 44 (38.60%) (Table 2).

## Discussion

In this study, we investigated the alpha thalassemia mutation in Rasht City in Gilan province (located in the north of Iran and southwest coast of the Caspian Sea). We identified 15 different mutations in this area. Gilan province has a diverse ethnic distribution; a majority of the population belong to the Gilaki ethnic group, followed by Talysh and Kurds (11).

As expected, we found that −α^3.7^ was the most common mutation among the study population, contributing to 48.24% of individuals; this finding is consistent with that of the previous studies in Iran (11–15). These results also align with those of neighboring countries (16–18). The second and third most common mutations found in our study were Hb C.S and poly A_2_, respectively. Our study results have several similarities with those of Hadavi et al. from Guilan, who found that −α^3.7^ (42.5%) was the most common mutation, followed by poly A_2_ (12.4%) and Hb C.S (10.6%), and those of Tamaddoni et al. from Mazandaran province (neighboring province), who showed that −α^3.7^ and poly A_2_ were the first and second common mutations (19). However, our finding is at variance with those of earlier studies conducted in Greece (20), Turkey (17), United Arab Emirates (21), and former research from Iran in 2003 (22), which demonstrated a low frequency of Hb C.S. This mutation had two different origins that caused Chinese and Mediterranean variants (11), so there was a possible gene flow in recent years from these two areas that resulted in an increasing frequency of Hb C.S.

Hb C.S is an α-chain variant caused by a point mutation; a base exchange (TAA-CAA) at the stop codon of the α_2_ globin gene resulted in an unstable α-globin mRNA and decreased α-globin chain production (23, 24). We identified one homozygous Hb C.S in our study without a history of blood transfusion and splenomegaly, which is a rare disorder in Western countries compared with Asia (25). Homozygous Hb C.S causes non-transfusion-dependent thalassemia without any signs of hepatosplenomegaly in adults, but it could cause severe anemia in the fetus that might slightly resolve after birth. The first case of hydrops fetalis due to homozygous Hb C.S was reported in 2006 in Thailand (26). Anemia during intrauterine life could result in serious health issues such as cardiovascular and metabolic disorders in adulthood because of hypoxia-related cellular damage (27–29). A case series study from Thailand reported six cases of patients with homozygous Hb C.S. The diagnosis was made by performing cordocentesis after the ultrasound anomaly scan indicated cardiomegaly, increased cardiothoracic diameter, high middle cerebral artery peak systolic velocity (MCA-PSV), and various degrees of hepatic and placental enlargement, and also some hydropic signs such as ascites. All these patients received intrauterine blood transfusion treatment; two patients had mild anemia after birth that resolved with phototherapy (30). Our results showed a high Hb C.S frequency, which contrasted with that of the previous study (22). The high prevalence of Hb C.S highlights the need for healthcare systems to pay significant attention to it because of the risk of Hb H disease caused by non-deletional mutations such as Hb C.S as well as intrauterine management approaches (30, 31).

We identified four patients with− −^MED^ in the study population. This mutation has a high prevalence in the Mediterranean area (32). Given the high rate of occurrence frequency of consanguineous marriages in Iran, we can expect an increased frequency of Hb H disease occurrence and possible hydrops fetalis.

Based on genetic mutations, most patients were recognized as silent carriers. They had normal Hb levels and mild hypochromic microcytic without anemia; as demonstrated in previous studies, most alpha thalassemia silent carriers either have mild anemia or their condition will be normal (33–35). It is possible to miss a diagnosis of Alpha thalassemia during life. Therefore, we expected a more broad distribution of alpha thalassemia mutations (3).

A high prevalence of alpha thalassemia mutations is found in the Mediterranean and Middle Eastern regions. Based on previous studies, it is found that up to 40% of these populations are carriers (36, 37). In this study, we found that the ethnic background of most of the identified mutations was Mediterranean and Middle Eastern mutation types. The type of alpha thalassemia mutations varies depending on geographic regions. However, our result is in tune with Iran’s geographic location (38).

## Conclusion

Iran is located in a high-prevalence alpha thalassemia geographic region. Due to cultural habits and customs and an increased frequency of consanguineous marriages, investigating mutation types and identifying alpha thalassemia carriers, as well as making a prenatal diagnosis of alpha thalassemia, have become critical to achieving better prevention and management.

## Data Availability

The original contributions presented in the study are included in the article/Supplementary Material, further inquiries can be directed to the corresponding author.

## References

[1] Modell B Darlison M. Global epidemiology of haemoglobin disorders and derived service indicators. Bull World Health Organ. (2008) 86:480–7. 10.2471/BLT.06.036673 18568278 PMC2647473

[2] Farashi S Harteveld CL. Molecular basis of α-thalassemia. Blood Cells Mol Dis. (2018) 70:43–53. 10.1016/j.bcmd.2017.09.004 29032940

[3] Harteveld CL Higgs DR. α-Thalassaemia. Orphanet J Rare Dis. (2010) 5:13. 10.1186/1750-1172-5-13 20507641 PMC2887799

[4] Moradi K Aznab M Tahmasebi S Dastafkan Z Omidniakan L Ahmadi M The spectrum of α-thalassemia mutations in the lak population of Iran. Hemoglobin. (2019) 43(2):107–11. 10.1080/03630269.2019.1614049 31304855

[5] Phylipsen M Prior JF Lim E Lingam N Vogelaar IP Giordano PC Thalassemia in western Australia: 11 novel deletions characterized by multiplex ligation-dependent probe amplification. Blood Cells Mol Dis. (2010) 44(3):146–51. 10.1016/j.bcmd.2009.12.011 20110179

[6] Mahdavi MR Hojjati MT Roshan P. A review on thalassemia and related complications. J Maz Univ Med Sci. (2013) 23(103):139–49.

[7] Fucharoen S Viprakasit V. Hb H disease: clinical course and disease modifiers. Hematology Am Soc Hematol Educ Program. (2009) 2009(1):26–34. 10.1182/asheducation-2009.1.26 20008179

[8] Vichinsky EP. Clinical manifestations of α-thalassemia. Cold Spring Harbor Perspect Med. (2013) 3(5):a011742. 10.1101/cshperspect.a011742 PMC3633183 23543077

[9] Dehbozorgian J Moghadam M Daryanoush S Haghpanah S Imani fard J Aramesh A Distribution of alpha-thalassemia mutations in Iranian population. Hematology. (2015) 20(6):359–62. 10.1179/1607845414Y.0000000227 25553732

[10] Samavat A Modell B. Iranian national thalassaemia screening programme. Br Med J. (2004) 329(7475):1134–7. 10.1136/bmj.329.7475.1134 15539666 PMC527686

[11] Hadavi V Jafroodi M Hafezi-Nejad N Moghadam SD Eskandari F Tarashohi S α-Thalassemia mutations in Gilan province, North Iran. Hemoglobin. (2009) 33(3–4):235–41. 10.1080/03630260903089029 19657838

[12] Harteveld C Yavarian M Zorai A Quakkelaar E Van Delft P Giordano P. Molecular spectrum of α-thalassemia in the Iranian population of hormozgan: three novel point mutation defects. Am J Hematol. (2003) 74(2):99–103. 10.1002/ajh.10385 14508795

[13] Gohari LH Petrou M Felekis X Christopoulos G Kleanthous M. Identification of α-thalassemia mutations in Iranian individuals with abnormal hematological indices and normal hb A2. Hemoglobin. (2003) 27(2):129–32. 10.1081/HEM-120021548 12779276

[14] Neishabury M Oberkanins C Moheb LA Pourfathollah AA Kahrizi K Keyhany E High prevalence of the −Α3. 7 deletion among thalassemia patients in Iran. Hemoglobin. (2003) 27(1):53–5. 10.1081/HEM-120018438 12603096

[15] Yavarian M Karimi M Zorai A Harteveld CL Giordano PC. Molecular basis of hb H disease in southwest Iran. Hemoglobin. (2005) 29(1):43–50. 10.1081/HEM-47019 15768554

[16] Khan SN Hasan F Sollaino C Perseu L Riazuddin S. Molecular characterization of alpha-thalassemia in Pakistan. Hemoglobin. (2003) 27(3):161–6. 10.1081/hem-120023379 12908800

[17] Karakaş Z Koç B Temurhan S Elgün T Karaman S Asker G Evaluation of alpha-thalassemia mutations in cases with hypochromic microcytic Anemia: the İstanbul perspective. Turk J Haematol. (2015) 32(4):344–50. 10.4274/tjh.2014.0204 26377141 PMC4805326

[18] Baysal E. α-Thalassemia syndromes in the United Arab Emirates. Hemoglobin. (2011) 35(5-6):574–80. 10.3109/03630269.2011.634698 22074123

[19] Tamaddoni A Hadavi V Nejad NH Khosh-Ain A Siami R Aghai-Meibodi J α-Thalassemia mutation analyses in Mazandaran province, North Iran. Hemoglobin. (2009) 33(2):115–23. 10.1080/03630260902817297 19373587

[20] Kanavakis E Papassotiriou I Karagiorga M Vrettou C Metaxotou-Mavrommati A Stamoulakatou A Phenotypic and molecular diversity of haemoglobin H disease: a Greek experience. Br J Haematol. (2000) 111(3):915–23. 10.1111/j.1365-2141.2000.02448.x 11122156

[21] El-Kalla S Baysal E. α-Thalassemia in the United Arab Emirates. Acta Haematol. (1998) 100(1):49–53. 10.1159/000040863 9691147

[22] Garshasbi M Oberkanins C Law HY Neishabury M Kariminejad R Najmabadi H. Alpha-globin gene deletion and point mutation analysis among in Iranian patients with microcytic hypochromic anemia. Haematologica. (2003) 88(10):1196–7. 10.3324/%x 14555321

[23] Clegg JB Weatherall DJ. Hemoglobin constant spring, an unusual α-chain variant involved in the etiology of hemoglobin H disease. Ann N Y Acad Sci. (1974) 232(1):168–78. 10.1111/j.1749-6632.1974.tb20582.x 4606609

[24] Morales J Russell JE Liebhaber SA. Destabilization of human α-globin mRNA by translation anti-termination is controlled during erythroid differentiation and is paralleled by phased shortening of the poly (a) tail. J Biol Chem. (1997) 272(10):6607–13. 10.1074/jbc.272.10.6607 9045690

[25] Laig M Pape M Hundrieser J Flatz G Sanguansermsri T Das B The distribution of the hb constant spring gene in southeast asian populations. Hum Genet. (1990) 84(2):188–90. 10.1007/BF00208939 2298455

[26] Charoenkwan P Sirichotiyakul S Chanprapaph P Tongprasert F Taweephol R Sae-Tung R Anemia and hydrops in a fetus with homozygous hemoglobin constant spring. J Pediatr Hematol Oncol. (2006) 28(12):827–30. 10.1097/01.mph.0000243662.56432.37 17164653

[27] Gillman M Rich-Edwards J. The fetal origin of adult disease: from sceptic to convert. Paediatr Perinat Epidemiol. (2000) 14(3):192–3. 10.1046/j.1365-3016.2000.00265.x 10949210

[28] Giussani DA Niu Y Herrera EA Richter HG Camm EJ Thakor AS Heart disease link to fetal hypoxia and oxidative stress. Adv Exp Med Biol. (2014) 814:77–87. 10.1007/978-1-4939-1031-1_7 25015802

[29] Shankaran S Das A Bauer CR Bada H Lester B Wright L Fetal origin of childhood disease: intrauterine growth restriction in term infants and risk for hypertension at 6 years of age. Arch Pediatr Adolesc Med. (2006) 160(9):977–81. 10.1001/archpedi.160.9.977 16953023

[30] Sirilert S Charoenkwan P Sirichotiyakul S Tongprasert F Srisupundit K Luewan S Prenatal diagnosis and management of homozygous hemoglobin constant spring disease. J Perinatol. (2019) 39(7):927–33. 10.1038/s41372-019-0397-7 31097760

[31] Sriiam S Leecharoenkiat A Lithanatudom P Wannatung T Svasti S Fucharoen S Proteomic analysis of hemoglobin H-constant spring (hb H-cs) erythroblasts. Blood Cells Mol Dis. (2012) 48(2):77–85. 10.1016/j.bcmd.2011.11.004 22154201

[32] Ko T-M Hwa H-L Liu C-W Li S-F Chu J-Y Cheung Y-P. Prevalence study and molecular characterization of α-thalassemia in filipinos. Ann Hematol. (1999) 78(8):355–7. 10.1007/s002770050528 10460348

[33] Singer ST. Variable clinical phenotypes of α-thalassemia syndromes. TheScientificWorldJournal. (2009) 9:615–25. 10.1100/tsw.2009.69 19618088 PMC5823233

[34] Kohne E. Hemoglobinopathies: clinical manifestations, diagnosis and treatment. Dtsch Arztebl Int. (2011) 108(31-32):532. 10.3238/arztebl.2011.0532 21886666 PMC3163784

[35] Rachmilewitz EA Giardina PJ. How I treat thalassemia. Blood. (2011) 118(13):3479–88. 10.1182/blood-2010-08-300335 21813448

[36] Vichinsky EP. Alpha thalassemia major – new mutations, intrauterine management, and outcomes. Hematology Am Soc Hematol Educ Program. (2009) 2009(1):35–41. 10.1182/asheducation-2009.1.35 20008180

[37] Weatherall DJ Clegg JB. Inherited haemoglobin disorders: an increasing global health problem. Bull World Health Organ. (2001) 79(8):704–12. PMID: ; PMCID: 11545326 PMC2566499

[38] Harteveld CL Traeger-Synodinos J Ragusa A Fichera M Kanavakis E Kattamis C Different geographic origins of hb constant spring [alpha (2) Codon 142 Taa–> Caa]. Haematologica. (2001) 86(1):36–8. 10.3324/%x 11146568

